# Clinical factors associated with adverse clinical outcomes in elderly versus non-elderly COVID-19 emergency patients: a multi-center observational study

**DOI:** 10.1186/s12245-023-00482-4

**Published:** 2023-02-22

**Authors:** Chanokporn Puchongmart, Phetsinee Boonmee, Supawich Jirathanavichai, Nutthida Phanprasert, Netiporn Thirawattanasoot, Thawonrat Dorongthom, Apichaya Monsomboon, Nattakarn Praphruetkit, Onlak Ruangsomboon

**Affiliations:** 1 Department of Emergency Medicine, Banphaeo General Hospital, Samutsakhon, Thailand; 2 Department of Emergency Medicine, Ratchaburi Hospital, Ratchaburi, Thailand; 3 Department of Emergency Medicine, Faculty of Medicine, Siriraj Hospital, Mahidol University, 2 Wanglang Road, Bangkoknoi, Bangkok, 10700 Thailand; 4 Department of Emergency and Forensic Medicine, Buddhachinaraj Phitsanulok Hospital, Phitsanulok, Thailand; 5 Department of Emergency and Forensic Medicine, Prachuap Khiri Khan Hospital, Prachuap Khiri Khan, Thailand

**Keywords:** COVID-19, Coronavirus, Elderly patients, Geriatric patients, Emergency department

## Abstract

**Background:**

The COVID-19 pandemic has caused over 6 million deaths worldwide. The elderly accounted for a large proportion of patients with their mortality rate largely higher than the non-elderly. However, limited studies have explored clinical factors associated with poor clinical outcomes in this important population. Therefore, this study aimed to determine factors independently associated with adverse clinical outcomes among COVID-19 elderly patients.

**Methods:**

We conducted a multicenter observational study at five emergency departments (EDs) in Thailand. Patients over 18 years old diagnosed with COVID-19 between January and December 2021 were included. We classified patients into elderly (age ≥ 65 years) and non-elderly (age < 65 years). The primary clinical outcome was in-hospital mortality. The secondary outcomes were endotracheal intubation and intensive care unit admission. We identified independent factors associating with these outcomes both in the whole population and separately by age group using multivariate logistic regression models.

**Results:**

A total of 978 patients were included, 519 (53.1%) were elderly and 459 (46.9%) were non-elderly, and 254 (26%) died at hospital discharge. The mortality rate was significantly higher in the elderly group (39.1% versus 14.3%, *p*<0.001)). In the elderly, age (adjusted odds ratio (aOR) 1.13; 95% confidence interval (CI) 1.1—1.2; *p*<0.001), male sex (aOR 3.64; 95%CI 1.5–8.8; *p*=0.004), do-not-resuscitate (DNR) status (aOR 12.46; 95%CI 3.8–40.7; *p*<0.001), diastolic blood pressure (aOR 0.96; 95%CI 0.9–1.0; *p*=0.002), body temperature (aOR 1.74; 95%CI 1.0–2.9; *p*=0.036), and Glasgow Coma Scale (GCS) score (aOR 0.71; 95%CI 0.5–1.0; *p*=0.026) were independent baseline and physiologic factors associated with in-hospital mortality. Only DNR status and GCS score were associated with in-hospital mortality in both the elderly and non-elderly, as well as the overall population. Lower total bilirubin was independently associated with in-hospital mortality in the elderly (aOR 0.34; 95%CI 0.1–0.9; *p*=0.035), while a higher level was associated with the outcome in the non-elderly. C-reactive protein (CRP) was the only laboratory factor independently associated with all three study outcomes in the elderly (aOR for in-hospital mortality 1.01; 95%CI 1.0–1.0; *p*=0.006).

**Conclusion:**

Important clinical factors associated with in-hospital mortality in elderly COVID-19 patients were age, sex, DNR status, diastolic blood pressure, body temperature, GCS score, total bilirubin, and CRP. These parameters may aid in triage and ED disposition decision-making in this very important patient population during times of limited resources during the COVID-19 pandemic.

**Supplementary Information:**

The online version contains supplementary material available at 10.1186/s12245-023-00482-4.

## Introduction

The global population is transitioning into an aging community. The world’s geriatric population has steadily risen as a result of increased life expectancy and declining birth rates. The United Nations projected that the number of individuals aged at least 65 years would nearly double in 2050, increasing from 10 to 22% [1]. Although aging represents normal physiological processes, it often results in frailty and increases adverse clinical outcomes in older people [2–4]. Age-related physiological decline affects most vital organs in cardiovascular, respiratory, renal, and immunological systems [4, 5]. Understanding their consequences is essential and will play a major role in future patient care in the light of the aging population.

The coronavirus disease 2019 (COVID-19) pandemic remains a significant concern worldwide in regard to disease control. The virus’ propensity to rapidly spread and mutate has caused a global rise in the number of infected patients despite various efficacious vaccine strategies [6]. There still remains a large volume of emergency department (ED) visits secondary to COVID-19, overwhelming most healthcare capacities [7]. Moreover, the number of deaths from COVID-19 is still increasing. The elderly, defined as patients aged 65 and above, accounted for 74% of all deaths, with a mortality rate 62 times higher than the younger population [8, 9]. They thus utilize a large volume of healthcare resources such as intensive care units (ICUs), hospitalizations, personal protective equipment, as well as healthcare providers [10, 11].

There have been several reported prognostic factors of poor outcomes among patients with COVID-19, for example, advancing age, obesity, various comorbidities, low blood pressure, hypoxemia, fever, and several laboratory abnormalities such as high white blood cells, low lymphocyte and platelet count, high D-dimer, high total bilirubin, and high C-reactive protein (CRP) [12–14]. However, to our knowledge, no studies have evaluated independent factors associated with adverse clinical outcomes, specifically in the elderly, who were at the highest risk of such outcomes, and compared with the non-elderly. Therefore, we aimed to determine factors associated with in-hospital mortality, endotracheal intubation, and ICU admission in elderly COVID-19 patients presenting to the ED. We also aimed to compare and contrast these prognostic factors among the elderly versus the non-elderly population.

## Methods

### Study design and setting

We conducted a multicenter, retrospective observational cohort study across five participating EDs in Thailand. Participating centers were Siriraj Hospital, Mahidol University, the largest university hospital in Bangkok (over 2000 in-patient beds); Banphaeo General Hospital, a large general hospital in Samut Sakhon province (350 in-patient beds); Ratchaburi Hospital, the main provincial and teaching hospital in Ratchaburi province (900 in-patient beds); Buddhachinnaraj Hospital, a tertiary regional advanced-level and teaching hospital in Phitsanulok province (900 in-patient beds); and Prachuap Kiri Kan Hospital, a general standard-level hospital of Prachuab Kiri Kan province (300 in-patient beds). Central Research Ethics Committee approved the study (certificate of approval COA-CREC044/2022). Patients’ informed consent was waived as per the retrospective nature of the study.

### Patients

We assessed all consecutive patients visiting the participating EDs from January 1, 2021, to December 31, 2021, during which the first large waves of COVID-19 attacked Thailand and large mass vaccinations for the whole population were advocated [15, 16]. Eligible patients were identified by using ICD-10 codes. They were enrolled if they aged at least 18 years and were diagnosed with SARS-CoV2 infection as confirmed by reverse transcription polymerase chain reaction (RT-PCR) performed within the index ED visit. Those with missing study outcomes were excluded. Following their inclusion, we classified them based on their age, those less than 65 years old as the non-elderly and those at least 65 years of age as the elderly.

### Data variables

Patients with confirmed SARS-CoV2 infection underwent laboratory investigation and received medical and non-medical treatment as per the Thai national guidelines for COVID-19 at the time of diagnosis. All participating hospitals strictly followed the national guidelines for evaluation, treatment, and disposition of COVID-19 patients. We extracted the following data from the patients’ medical records: age, gender, body mass index (BMI), comorbidities, day of symptoms upon ED visit, vital signs and mental status at ED arrival, first laboratory results taken in the ED or within the first day of admission, COVID-19 specific and supportive management, complications, ED disposition, and important clinical outcomes.

### Study objectives and outcomes

The primary objective was to identify independent factors associated with poor clinical outcomes in the elderly compared to the non-elderly population. The primary clinical outcome of interest was in-hospital mortality. The secondary outcomes were endotracheal intubation and ICU admission.

### Statistical analysis

We reported patients’ characteristics as frequency (percentage) and compared them using chi-squared or Fisher’s exact test for categorical variables. Continuous variables are described as mean and standard deviation or median and interquartile range and were compared using Student’s *T*-test or Mann–Whitney *U* test, respectively, as appropriate. Patient characteristics are presented and compared by mortality status at hospital discharge and also by elderly status.

Independent factors associated with each adverse clinical outcome of interest were identified by using multivariate logistic regression analyses. Because we found a non-significant within-cluster effect by the study center from running a generalized linear mixed model (*p*=0.233), we instead employed multivariate logistic regression analyses using the backward stepwise method to identify independent prognostic factors. Potential associated factors included in the first step were variables that could have predicted each study outcome. Variables in the first model for in-hospital mortality included baseline demographics, initial vital signs, laboratory results, management, and complications. For endotracheal intubation and ICU admission, management and complications were not included in the regression models as these variables could have occurred after the outcomes. Results from the final model were reported as adjusted odds ratio (aOR) and 95% confidence interval (95% CI) with its associated *p*-value. We estimated that we would have acquired enough primary outcome events to reach the minimum of 10 events per variable for the logistic regression analyses from the 1-year data. There were less than 10% missing data for all variables, so they were considered to be missing at random, and no maneuvers were employed to handle them. The analyses were performed both in the whole study population and separately by their elderly status. All analyses were performed using SPSS 18.0 (IBM Corp., Chicago, IL).

## Results

### Characteristics of patients

A total of 978 patients diagnosed with SARS-CoV2 infection visited the five participating EDs from January 1, 2021, to December 31, 2021 and were included. No patients were excluded since all study outcomes could be retrieved. Their baseline characteristics by mortality status are presented in Table 1. The mean age of the study population was 62.3 years, and 50.3% of them were male. Of the included patients, 254 (26%) died at hospital discharge, 155 (15.8%) required endotracheal intubation, and 95 (9.8%) were admitted to the ICU (Table 1). Patients with in-hospital mortality were significantly older, had more underlying comorbidities, more severe abnormal vital signs and laboratory results, and had more complications than those discharged alive.

**Table 1:** Patient characteristics by mortality status at hospital discharge

Characteristics	All patients (*n*=978)	Dead (*n*=254)	Alive (*n*=724)	*p*-value
Age	62.30±18.1	72.91±15.1	58.59±17.6	<0.001
Sex (male)	492 (50.3)	149 (58.7)	343 (47.4)	0.002
Body mass index	25.86±6.27	25.12±6.70	26.12±6.10	0.032
Day of symptoms	4, 5	4, 3	4, 5	0.001
Underlying conditions				
Coronary artery disease	90 (9.2)	34 (13.4)	56 (7.7)	0.007
Cerebrovascular disease	92 (9.4)	40 (15.7)	52 (7.2)	<0.001
Chronic pulmonary disease	81 (8.3)	29 (11.4)	52 (7.2)	0.035
Diabetes mellitus	323 (33)	86 (33.9)	237 (32.7)	0.743
Moderate to severe renal disease	145 (14.8)	63 (24.8)	82 (11.3)	<0.001
Cancer	64 (6.5)	23 (9.1)	41 (5.7)	0.060
Immunodeficiency status	30 (3.1)	9 (3.5)	21 (2.9)	0.609
Do-not-resuscitate status	228 (23.3)	163 (64.2)	65 (9.0)	<0.001
Charlson comorbidity index	1, 3	2, 4	1, 2	<0.001
Vital signs and mental status				
Systolic blood pressure (mmHg)	136.53±28.95	136.64±30.20	136.49±28.5	0.942
Diastolic blood pressure (mmHg)	78.66±16.61	75.90±18.8	79.63±15.7	0.002
Pulse rate (beats/min)	95.71±19.07	98.94±21.4	94.58±19.0	0.002
Respiratory rate (breaths/min)	28, 12	32, 10	28, 10	<0.001
Body temperature (°C)	36.9, 1.0	37.0, 1.2	36.9, 1.0	0.020
Glasgow Coma Scale score	15, 0	15, 1	15, 0	<0.001
Oxygen saturation (%)	92, 11	88, 17	94, 7	<0.001
Laboratory results				
Hemoglobin (g/dL)	12.5, 2.8	12.2, 3.3	12.6, 2.6	0.013
White blood cells (×10^3^/μL)	7.2, 4.8	7.3, 5.5	6.9, 4.5	0.012
Platelet (×10^4^/μL)	21.6, 10.8	22.2, 11	20.3, 10.3	<0.001
Glomerular filtration rate (ml/min/1.73m^2^)	64.65±33.71	52.08±29.4	74.53±33.5	<0.001
Lactate (mg/dL)	0.52±1.51	1.71±2.9	0.42±1.0	<0.001
D-dimer (g/L)	0.67, 1.69	0.961, 2.11	0.50, 1.24	<0.001
Aspartate transaminase (AST) (mg/dL)	44, 39	52, 50	42, 38	<0.001
Alanine aminotransferase (ALT) (mg/dL)	26, 27	28, 31	27, 33	0.717
Total bilirubin (mg/dL)	0.44, 0.36	0.50, 0.43	0.47, 0.34	0.128
Procalcitonin (mg/L)	0.20, 0.55	0.36, 1.31	0.15, 0.19	<0.001
C-reactive protein (μg/L)	69.16, 87.4	82.4, 121.35	60.88, 90.84	<0.001
Management				
Corticosteroids	841 (86.2)	242 (95.3)	599 (83.0)	<0.001
Favipiravir	930 (95.2)	238 (93.7)	692 (95.7)	0.197
Remdesivir	145 (14.8)	56 (22.0)	89 (12.3)	<0.001
Tocilizumab	54 (5.5)	18 (7.1)	36 (5.0)	0.206
Baricitinib	12 (1.2)	5 (2.0)	7 (1.0)	0.213
High flow nasal cannula	173 (17.7)	63 (24.8)	110 (15.2)	0.001
Endotracheal intubation	155 (15.8)	109 (42.9)	46 (6.4)	<0.001
Extracorporeal membrane oxygenation	2 (0.2)	2 (0.8)	0 (0.0)	0.017
Inotropic drugs	140 (14.3)	108 (42.5)	32 (4.4)	<0.001
Renal replacement therapy	45 (4.6)	20 (7.9)	25 (3.5)	0.004
Emergency department disposition				
ICU	95 (9.8)	41 (16.2)	54 (7.5)	<0.001
Intermediate ward	506 (52.1)	157 (62.1)	349 (48.5)	<0.001
Low-acuity ward	254 (26.1)	25 (9.9)	229 (31.8)	<0.001
Field hospital	44 (4.5)	1 (0.4)	43 (6.0)	<0.001
^a^Hospitel	2 (0.2)	0 (0.0)	2 (0.3)	<0.001
Home isolation	32 (3.3)	0 (0.0)	32 (4.5)	<0.001
Transfer	8 (0.8)	0 (0.0)	8 (1.1)	<0.001
Dead	29 (3.0)	29 (3.0)	0 (0.0)	<0.001
Other	2 (0.2)	0 (0.0)	2 (0.3)	<0.001
Complications				
Ventilator associated pneumonia	39 (4.0)	24 (9.4)	15 (2.1)	<0.001
Hospital associated pneumonia	125 (12.8)	80 (31.5)	45 (6.2)	<0.001
Bacterial pneumonia	170 (17.4)	92 (36.2)	78 (10.8)	<0.001
Other hospital-acquired infection	89 (9.1)	38 (15.0)	51 (7.0)	<0.001
Septic shock	137 (14.0)	103 (40.6)	34 (4.7)	<0.001
Acute respiratory distress syndrome	146 (14.9)	104 (40.9)	42 (5.8)	<0.001
Pulmonary embolism	29 (3)	13 (5.1)	16 (2.2)	0.019
Stroke	6 (0.6)	4 (1.6)	2 (0.3)	0.023
Myocardial infarction	7 (0.7)	7 (2.8)	0 (0.0)	<0.001
Pneumothorax	20 (2.0)	11 (4.3)	9 (1.2)	0.003
Outcomes				
Hospital length of stay (days)	10, 9	10, 12	10, 8	0.424
ICU length of stay (days)	8, 10	10, 10	7, 5	0.003

Note: data presented as *n* (%), mean±standard deviation or median, interquartile range

*ICU* intensive care unit, *mmHg* millimeters of mercury, *mm*^*2*^ square millimeters

^a^Hotels that were modified into hospitals for low-acuity patients

After age stratification, 519 (53.1%) were categorized as the elderly, while 459 (46.9%) were in the non-elderly group. The elderly generally had more comorbidities and complications, as shown in Table 2. Also, the mortality rate was significantly higher in the elderly (39.1%) compared to the non-elderly (14.3%) (*p*<0.001). However, the rate of intubation, high-flow nasal cannula (HFNC) utilization, renal replacement therapy, ICU admission, and ICU and hospital length of stay were not significantly different between the two groups. They also received similar treatment except for a higher rate of steroid and inotropic drugs prescription in the elderly (Table 2). Patients’ characteristics by study center are presented in Table S1 in the Supplementary Additional file 1.

**Table 2:** Patient characteristics by age group (elderly versus non-elderly)

Characteristics	Age < 65 years (*n*=459)	Age > 65 years (*n*=519)	*p*-value
Age	48.76±13.0	77.63±7.9	n/a
Sex (male)	245 (53.4)	247 (47.6)	0.098
Body mass index	27.37±6.9	24.20±5.1	<0.001
Day of symptoms	4, 5	3, 4	0.011
Underlying conditions			
Coronary artery disease	27 (5.2)	63 (13.7)	<0.001
Cerebrovascular disease	22 (4.2)	70 (15.3)	<0.001
Chronic pulmonary disease	34 (6.6)	47 (10.2)	0.037
Diabetes mellitus	137 (26.4)	186 (40.5)	<0.001
Moderate to severe renal disease	46 (8.9)	99 (21.6)	<0.001
Cancer	19 (3.7)	45 (9.8)	<0.001
Immunodeficiency status	21 (4.0)	9 (2.0)	0.059
Do-not-resuscitate status	17 (3.3)	32 (7.0)	0.008
Charlson comorbidity index	0, 1	2, 4	<0.001
Vital signs and mental status			
Systolic blood pressure (mmHg)	134.48±27.8	138.84±30.0	0.019
Diastolic blood pressure (mmHg)	80.90±16.8	76.13±16.1	<0.001
Pulse rate (beats/min)	96, 24	92, 28	0.011
Respiratory rate (breaths/min)	28, 10	30, 12	0.005
Body temperature (°C)	36.8, 1.0	37.0, 1.1	0.049
Glasgow Coma Scale score	15, 0	15, 0	<0.001
Oxygen saturation (%)	94, 8	92, 10	<0.001
Laboratory results			
Hemoglobin (g/dL)	12.9, 2.5	12.1, 2.9	0.001
White blood cells (×10^3^/μL)	6.9, 4.7	7.1, 4.5	0.247
Platelet (×10^4^/μL)	22.5, 11.5	20.8, 10.4	0.016
Creatinine (mg/dL)	1.51±2.3	1.53±1.5	
Glomerular filtration rate (ml/min/1.73m^2^)	81.20±34.8	54.95±26.9	<0.001
Lactate (mg/dL)	0.61±1.88	1.00±1.90	<0.001
D-dimer (g/L)	0.45, 1.08	0.87, 2.06	<0.001
Aspartate transaminase (AST) (mg/dL)	45, 39	43, 42	0.342
Alanine aminotransferase (ALT) (mg/dL)	34, 35	24, 25	<0.001
Total bilirubin (mg/dL)	0.47, 0.37	0.47, 0.35	0.278
Procalcitonin (mg/L)	0.16, 0.37	0.21, 0.66	<0.001
C-reactive protein (μg/L)	61.98, 94.46	69.96, 101.76	0.045
Management			
Corticosteroids	419 (80.9)	422 (92.1)	<0.001
Favipiravir	490 (94.6)	440 (95.9)	0.356
Remdesivir	66 (12.7)	79 (17.2)	0.050
Tocilizumab	31 (6.0)	23 (5.0)	0.506
Baricitinib	6 (1.2)	6 (1.3)	0.833
High flow nasal cannula	86 (16.6)	87 (19)	0.330
Intubation	78 (15.0)	77 (16.8)	0.455
Extracorporeal membrane oxygenation	1 (0.2)	1 (0.2)	0.932
Inotropic drugs	54 (10.4)	86 (18.7)	<0.001
Renal replacement therapy	27 (5.2)	18 (3.9)	0.337
Emergency department disposition			
ICU	97 (18.7)	88 (19.2)	0.848
Intermediate ward	230 (44.7)	276 (60.4)	<0.001
Low-acuity ward	169 (32.8)	85 (18.6)	<0.001
Field hospital	29 (5.6)	15 (3.3)	<0.001
^a^Hospitel	2 (0.4)	0 (0.0)	<0.001
Home isolation	19 (3.7)	13 (2.8)	<0.001
Transfer	8 (1.6)	0 (0.0)	<0.001
Dead	6 (1.2)	23 (5.0)	<0.001
Other	2 (0.4)	0 (0.0)	<0.001
Complications			
Ventilator-associated pneumonia	18 (3.5)	21 (4.6)	0.377
Hospital-associated pneumonia	49 (9.4)	76 (16.6)	0.001
Bacterial pneumonia	64 (12.3)	106 (23.1)	<0.001
Other hospital-acquired infection	30 (5.8)	59 (12.9)	<0.001
Septic shock	52 (10.0)	85 (18.5)	<0.001
Acute respiratory distress syndrome	61 (11.8)	85 (18.5)	0.003
Pulmonary embolism	13 (2.5)	16 (3.5)	0.367
Stroke	1 (0.2)	5 (1.1)	0.073
Myocardial infarction	2 (0.4)	5 (1.1)	0.192
Pneumothorax	8 (1.5)	12 (0.6)	0.237
Outcomes			
Hospital length of stay (days)	10, 9	10, 9	0.474
ICU length of stay (days)	7, 8	9, 10	0.085
Hospital mortality	74 (14.3)	180 (39.2)	<0.001

Note: data presented as *n* (%), mean±standard deviation or median, interquartile range

*ICU* intensive care unit, *mmHg* millimeters of mercury, *mm*^*2*^ square millimeters

^a^Hotels that were modified into hospitals for low-acuity patients

### Factors associated with adverse outcomes

The full lists of variables included in the first step of the multivariate logistic regression models are elaborated in the footnotes of Tables 3, 4 and 5. Independent factors associated with in-hospital mortality in the whole population, the elderly, and the non-elderly groups were study center, do-not-resuscitate (DNR) status, and mental status assessed with Glasgow Coma Scale (GCS) (Table 3). Patients presenting to most centers other than Siriraj Hospital, the largest study center, tended to have higher odds of mortality compared to Siriraj Hospital patients. DNR status and impaired mental status were also associated with in-hospital mortality in all models of the three populations. Age (aOR 1.13; 95%CI 1.1–1.2; *p*<0.001), male sex (aOR 3.64; 95%CI 1.5–8.8; *p*=0.004), blood pressure (aOR 0.96; 95%CI 0.9–1.0; *p*=0.002), body temperature (aOR 1.74; 95%CI 1.0–2.9; *p*=0.036), and C-reactive protein (CRP) (aOR 1.01; 95%CI 1.0–1.0; *p*=0.006) were only associated with in-hospital mortality in the elderly but not in the non-elderly group. In contrast, a higher Charlson comorbidity index, lower oxygen saturation, higher procalcitonin, and the use of HFNC were associated with in-hospital mortality in the non-elderly but not the elderly group. Lower total bilirubin was associated with in-hospital mortality in the elderly group (aOR 0.34; 95%CI 0.1–0.9; *p*=0.035); in contrast, a higher level was associated with the same outcome in the non-elderly group. In the model with the whole population, many complications were also associated with in-hospital mortality, as well as other abnormal laboratory results, namely platelet, renal function, and lactate (Table 3).

**Table 3:** Multivariate analyses of factors associated with mortality in all patients and the elderly versus non-elderly

Factors	All patients (aOR, 95%CI)	***p***-value	Non-elderly (aOR, 95%CI)	***p***-value	Elderly (aOR, 95%CI)	***p***-value
Center	-	0.046	-	0.007	-	0.091
Siriraj Hospital	Reference	-	Reference	-	Reference	-
Banphaeo General Hospital	3.07 (0.0-303.0)	0.632	0.00 (0.0-0.0)	1.000	0.00 (0.0-0.0)	0.999
Ratchaburi Hospital	13.46 (1.9-95.4)	0.009	8.38 (2.3-30.0)	0.001	9.61 (1.3-72.7)	0.028
Prachuap Kiri Khan Hospital	3.31 (0.6-18.5)	0.174	4.26 (1.1-17.0)	0.040	-^a^	-^a^
Buddhachinaraj Hospital	-^a^	-^a^	0.88 (0.1-8.1)	0.908	-^a^	-^a^
Age	1.06 (1.0-1.1)	0.001	n/a	n/a	1.13 (1.1-1.2)	<0.001
Male sex	2.50 (1.0-6.1)	0.043	n/a	n/a	3.64 (1.5-8.8)	0.004
Do-not-resuscitate status	6.14 (1.4-27.2)	0.017	34.34 (5.9-200.2)	<0.001	12.46 (3.8-40.7)	<0.001
Charlson comorbidity index	n/a	n/a	1.26 (1.1-1.5)	0.004	n/a	n/a
Systolic blood pressure	1.02 (1.0-1.0)	0.037	n/a	n/a	n/a	n/a
Diastolic blood pressure	0.95 (0.9-1.0)	0.029	n/a	n/a	0.96 (0.9-1.0)	0.002
Body temperature	n/a	n/a	n/a	n/a	1.74 (1.0-2.9)	0.036
Oxygen saturation	n/a	n/a	0.95 (0.9-1.0)	0.006	n/a	n/a
Glasgow Coma Scale score	0.79 (0.6-1.0)	0.074	0.74 (0.6-0.9)	0.016	0.71 (0.5-1.0)	0.026
Platelet	1.0 (1.0-1.0)	0.014	n/a	n/a	n/a	n/a
Glomerular filtration rate	0.98 (1.0-1.0)	0.034	n/a	n/a	n/a	n/a
Lactate	1.68 (1.2-2.4)	0.003	n/a	n/a	n/a	n/a
C-reactive protein	n/a	n/a	n/a	n/a	1.01 (1.0-1.0)	0.006
Procalcitonin	n/a	n/a	1.03 (1.0-1.1)	0.085	n/a	n/a
Total bilirubin	n/a	n/a	1.51 (1.1-2.1)	0.016	0.34 (0.1-0.9)	0.035
Alanine aminotransferase	n/a	n/a	0.99 (1.0-1.0)	0.308	n/a	n/a
Renal replacement therapy	0.13 (0.0-0.8)	0.030	n/a	n/a	n/a	n/a
High flow nasal cannula	n/a	n/a	2.14 (0.8-5.7)	0.131	n/a	n/a
Intubation	7.80 (1.0-58.0)	0.045	n/a	n/a	n/a	n/a
Hospital associated pneumonia	4.37 (1.6-11.7)	0.003	n/a	n/a	n/a	n/a
Septic shock	10.20 (3.0-34.0)	<0.001	n/a	n/a	n/a	n/a
Acute respiratory distress syndrome	36.04 (8.8-148.4)	<0.001	n/a	n/a	n/a	n/a
Pulmonary embolism	0.08 (0.0-0.8)	0.031	n/a	n/a	n/a	n/a
Pneumothorax	0.10 (0.0-1.1)	0.056	n/a	n/a	n/a	n/a
ICU admission	0.03 (0.0-0.2)	<0.001	n/a	n/a	n/a	n/a

Variables included in the model were study center, age, sex, body mass index, day of symptoms, Charlson comorbidity index, do-not-resuscitate status, body temperature, systolic blood pressure, diastolic blood pressure, pulse rate, respiratory rate, oxygen saturation, Glasgow Coma Scale score, hemoglobin, white blood cells, platelet, glomerular filtration rate, D-dimer, lactate, C-reactive protein, total bilirubin, aspartate transaminase, alanine aminotransferase, procalcitonin, high-flow nasal cannula, steroid, favipiravir, remdesivir, tocilizumab, baricitinib, extracorporeal membrane oxygenation, inotropic drugs, renal replacement therapy, ICU admission, and complications

*aOR* adjusted odds ratio, *CI* confidence interval, *ICU* intensive care unit

^a^Inadequate sample size

**Table 4:** Multivariate analyses of factors associated with endotracheal intubation in all patients and the elderly versus non-elderly

Factors	All patients (aOR, 95%CI)	***p***-value	Non-elderly (aOR, 95%CI)	***p***-value	Elderly (aOR, 95%CI)	***p***-value
Age	n/a	n/a	n/a	n/a	0.93 (0.9-1.0)	0.021
Male sex	1.88 (1.0-3.7)	0.061	n/a	n/a	n/a	n/a
Day of symptoms	0.91 (0.8-1.0)	0.098	n/a	n/a	n/a	n/a
Body mass index	1.05 (1.0-1.1)	0.038	1.07 (1.0-1.2)	0.044	n/a	n/a
Systolic blood pressure	1.01 (1.0-1.0)	0.059	n/a	n/a	1.02 (1.0-1.0)	0.019
Pulse rate	1.02 (1.0-1.0)	0.016	n/a	n/a	1.03 (1.0-1.1)	0.005
Body temperature	n/a	n/a	n/a	n/a	2.05 (1.2-3.5)	0.006
Respiratory rate	1.04 (1.0-1.1)	0.078	n/a	n/a	n/a	n/a
Oxygen saturation	0.94 (0.9-1.0)	<0.001	0.91 (0.9-1.0)	<0.001	0.95 (0.9-1.0)	0.021
Glasgow Coma Scale score	0.81 (0.7-1.0)	0.015	0.71 (0.5-1.1)	0.111	0.76 (0.6-1.0)	0.028
Glomerular filtration rate	0.99 (1.0-1.0)	0.101	0.99 (1.0-1.0)	0.063	n/a	n/a
Platelet	n/a	n/a	1.00 (1.0-1.0)	0.093	n/a	n/a
Alanine aminotransferase	n/a	n/a	0.98 (1.0-1.0)	0.042	n/a	n/a
C-reactive protein	n/a	n/a	n/a	n/a	1.0 (1.0-1.0)	0.013
High flow nasal cannula	2.70 (1.3-5.4)	0.005	6.25 (2.3-17.0)	<0.001	n/a	n/a

Variables included in the model were study center, age, sex, body mass index, day of symptoms, Charlson comorbidity index, body temperature, systolic blood pressure, diastolic blood pressure, pulse rate, respiratory rate, oxygen saturation, Glasgow Coma Scale score, hemoglobin, white blood cells, platelet, glomerular filtration rate, D-dimer, lactate, C-reactive protein, total bilirubin, aspartate transaminase, alanine aminotransferase, procalcitonin, and high-flow nasal cannula. Do-not-resuscitate status was not included in the model because there was no outcome events in those declined to be intubated.

*aOR* adjusted odds ratio, *CI* confidence interval

**Table 5:** Multivariate analyses of factors associated with ICU admission in in all patients and the elderly versus non-elderly

Factors	All patients (aOR, 95%CI)	***p***-value	Non-elderly (aOR, 95%CI)	***p***-value	Elderly (aOR, 95%CI)	***p***-value
Center	n/a	n/a	n/a	n/a	-	0.049
Siriraj Hospital					Reference	-
Banphaeo General Hospital					0.00 (0.0-0.0)	0.999
Ratchaburi Hospital					10.82 (1.6-72.6)	0.014
Buddhachinaraj Hospital					-^a^	-^a^
Prachuap Kiri Khan Hospital					-^a^	-^a^
Age	0.98 (1.0-1.0)	0.014	n/a	n/a	0.90 (0.8-1.0)	0.006
Sex	n/a	n/a	n/a	n/a	2.43 (0.9-6.7)	0.087
Do-not-resuscitate status	3.14 (1.3-7.3)	0.008	n/a	n/a	10.52 (3.1-35.8)	<0.001
Systolic blood pressure	1.01 (1.0-1.0)	0.041	n/a	n/a	1.03 (1.0-1.0)	0.002
Diastolic blood pressure	n/a	n/a	n/a	n/a	n/a	n/a
Pulse rate	1.02 (1.0-1.0)	0.050	n/a	n/a	1.02 (1.0-1.0)	0.057
Body temperature	1.39 (1.0-1.9)	0.044	n/a	n/a	n/a	n/a
Oxygen saturation	0.96 (0.9-1.0)	0.001	0.92 (0.9-1.0)	<0.001	0.95 (0.9-1.0)	0.021
Hemoglobin	n/a	n/a	n/a	n/a	0.73 (0.6-0.9)	0.012
White blood cell count	1.00 (1.0-1.0)	0.098	n/a	n/a	n/a	n/a
Platelet	n/a	n/a	1.00 (1.0-1.0)	0.022	n/a	n/a
Lactate	1.48 (1.2-1.9)	0.002	1.51 (1.0-2.3)	0.064	1.58 (1.0-2.4)	0.034
D-dimer	n/a	n/a	n/a	n/a	1.0 (1.0-1.0)	0.023
C-reactive protein	1.0 (1.0-1.0)	0.032	n/a	n/a	1.0 (1.0-1.0)	0.013
High-flow nasal cannula	4.09 (2.2-7.7)	<0.001	6.58 (2.8-15.5)	<0.001	n/a	n/a

Variables included in the model were study center, age, sex, body mass index, day of symptoms, Charlson comorbidity index, do-not-resuscitate status, body temperature, systolic blood pressure, diastolic blood pressure, pulse rate, respiratory rate, oxygen saturation, Glasgow Coma Scale score, hemoglobin, white blood cells, platelet, glomerular filtration rate, D-dimer, lactate, C-reactive protein, total bilirubin, aspartate transaminase, alanine aminotransferase, procalcitonin, and high-flow nasal cannula

*aOR* adjusted odds ratio, *CI* confidence interval

^a^Inadequate sample size

For endotracheal intubation, impaired oxygen saturation and lower GCS were associated with the outcome in all three populations (Table 4). No other vital signs were associated with intubation in the non-elderly, while systolic blood pressure (aOR 1.02; 95%CI 1.0–1.0; *p*=0.019), pulse rate (aOR 1.03; 95%CI 1.0–1.1; *p*=0.005), and body temperature (aOR 2.05; 95%CI 1.2–3.5; *p*=0.006) were independent factors in the elderly group. Furthermore, higher BMI and HFNC use were strongly associated with intubation in the whole population and the non-elderly but not in the elderly group, while higher CRP (aOR 1.01; 95%CI 1.0–1.0; *p*=0.006) was only associated with intubation in the elderly population (Table 4).

For ICU admission, lower oxygen saturation and higher lactate were independently associated with the outcome in all three models (Table 5). Similar to the results for intubation, HFNC use was only associated with ICU admission in the overall and non-elderly model but not in the elderly one. There were more independent factors in the elderly than the non-elderly group, such as age (aOR 0.9; 95%CI 0.8–1.0; *p*=0.006), D-dimer (aOR 1.0; 95%CI 1.0–1.0; *p*=0.023), and CRP (aOR 1.0; 95%CI 1.0–1.0; *p*=0.013) (Table 5).

## Discussion

This study was a multicenter study evaluating prognostic factors of adverse clinical outcomes of COVID-19 in the elderly compared to non-elderly patients. We found that the elderly accounted for over 50% of all the included patients, reflecting the burden this population has on the healthcare system. Moreover, the elderly group was found to have approximately 2.7 times higher mortality rate than the non-elderly group. Although this direction of effect was concordant with the current evidence, the magnitude of difference in mortality rate among the two age groups in the present study was largely lower than a previous study by Yanez et al., who reported a 62-times higher mortality in the elderly group [8]. This discordance might have resulted from a paradigm shift of COVID-19 from a natural disaster at the beginning of the pandemic, from which the previous study was derived, when vaccines and healthcare resources were critically limited, as compared to our study that included patients from a subsequent period, during which the disease has become an endemic with improving healthcare resources utilization and management guidelines. As a result, the present study could have revealed the actual mortality rate among the elderly with the current pandemic situation now gradually resolving.

Interestingly, we found that the odds of in-hospital mortality among other institutions were higher than that of the largest study center in Bangkok. This result could have been explained by the differential quality of treatment and availability of healthcare resources and healthcare personnel with adequate expertise, most of which were accumulated in large and central institutions during the early and middle phases of the outbreak. The finding thus highlights the potential equity issue we experienced during the pandemic, which should be a concern for the government or other responsible departments should future healthcare catastrophes occur.

Although many previous studies have evaluated factors predicting adverse outcomes in the elderly population [17], none compared the results between the elderly and the non-elderly. Therefore, this study adds to the body of evidence the different characteristics and predictors of outcomes among the two populations. We observed that in-hospital mortality was associated with abnormal vital signs and comorbidities in the elderly, concordant with many previous studies [17–20]. However, from our results, GCS seemed to be the strongest vital variable among all others with the highest strength of association in all models, not only for in-hospital mortality but also for endotracheal intubation. Furthermore, among laboratory results that were independently associated with adverse outcomes in the elderly, elevated CRP was found to be associated with all important clinical outcomes (Youden index cut-point of 106 mg/L), similar to previous studies [17, 20]. Serum CRP has been proven to be one of the strong inflammatory markers reflecting immunologic complications in severe SARS-CoV2 infection [21–24]. According to a previous study by Villoteau et al., elevated CRP was associated with the need for respiratory support, ICU admission and mortality in COVID-19 patients [25]. The result of our study thus confirms the importance of CRP as a marker of disease severity and adds to the body of evidence that CRP is also an independent clinical factor and a potential predictor of poor outcomes of COVID-19 in the elderly population. Another interesting laboratory result was total bilirubin level. Previous studies have shown that an increased total bilirubin value was associated with adverse outcomes in COVID-19 patients [13, 26]. Although we found concordant results among the non-elderly, lower total bilirubin was, in contrast, associated with in-hospital mortality in the elderly. This contrasting finding might have been because of the increased oxidative stress associated with people with advancing age and their ability to produce total bilirubin, a known strong anti-oxidant [27]. Many studies have demonstrated that the elderly with lower total bilirubin levels were at higher risk of adverse outcomes [27, 28]. Our result thus supports that such a circumstance is also true in COVID-19 elderly patients.

Moreover, in the present study, impaired oxygenation and HFNC use were only associated with in-hospital mortality in the non-elderly but not in the elderly, which may imply that the respiratory system probably was the leading cause of mortality in the non-elderly, whereas in the elderly, there probably was multi-organ system failure or other systemic consequences leading to mortality, as demonstrated by high body temperature and low diastolic blood pressure associating with in-hospital mortality only in the elderly group. However, this was only our speculation, and no other studies have demonstrated similar results. Therefore, further studies are required to confirm these findings and strengthen our interpretations.

For endotracheal intubation, we found that hypoxemia was associated with the outcome in all populations, similar to most previous studies [15, 29]. However, respiratory rate was not associated with intubation in both the non-elderly and elderly groups despite being a significant factor in the overall population. This result may reflect the pathophysiology of COVID-19, from which patients usually present with “silent hypoxemia”. Additionally and interestingly, the use of HFNC was very strongly associated with intubation, as well as ICU admission, in the non-elderly but not in the elderly population despite a similar proportion of HFNC application in both groups. This could mean there was less HFNC failure or HFNC might have been used mainly for palliative purposes in the elderly population. Regardless, when compared to other previous studies, our results were mostly dissimilar to theirs, probably because factors associated with intubation in COVID-19 patients varied between different settings with different management guidelines. Nonetheless, one concordant finding between other previous studies and the overall population in the present study was male sex associating with a higher risk of intubation [30, 31]. However, in contrast to those previous studies, we found that advancing age was not associated with intubation. In fact, in our elderly population, the older the patients were, the lower risk they had of being intubated. This result could have also reflected the higher tendency to choose palliative management strategies resulting in the decision not to intubate very elderly patients in our population.

We also discovered that, unsurprisingly, hypoxemia and increased lactate were strongly associated with ICU admission in all three study populations. In the non-elderly group, HFNC use was the strongest factor associating with ICU admission, with only a small number of other significant laboratory factors. On the contrary, there were many other physiologic variables and laboratory markers associating with this outcome in the elderly. These results again may reflect the likelihood of the elderly having more organ failure other than the respiratory system, which led to higher disease severity and thus prompted the decision to admit to the ICU. Moreover, in addition to CRP, D-dimer has been another blood marker that can be elevated in COVID-19 and has proven to prognosticate adverse outcomes accurately [32, 33]. Concordantly, in the present study, we found that a higher D-dimer level was independently associated with ICU admission in the elderly, similar to previous studies [32, 33].

### Limitation

There were some limitations to the present study. First, although the study was a multicenter study involving many EDs of hospitals with various levels of care, most of the included patients were from large tertiary-care centers, which may still limit the study’s generalizability. Second, we only included patients with confirmed COVID-19 known within the index ED visit. Therefore, we could have missed some eligible patients who did not receive COVID-19 confirmatory testing or were mis-triaged upon the index visit. Third, we started collecting data from the first large waves of the pandemic in the country, during which healthcare resources and hospital capacity available for endotracheal intubation and ICU admission could have been extremely limited, thus affecting the credibility of the independent factors from the regression models. Fourth, due to the retrospective nature of the study, we could not accurately obtain clinical scores specific to the elderly population, such as the clinical frailty score (CFS) and the Identification of Seniors at Risk (ISAR) score. Lastly, in some regression models, especially for the secondary clinical outcomes in which the outcome events were smaller than that of the primary, there might not have been adequate number of events per variable in the regression models, probably resulting in model overfitting. Further prospective studies enrolling patients from a more representative population with adequate sample size and event rates for all regression models should be conducted to confirm and strengthen the results of the present study.

## Conclusion

Important independent factors associated with in-hospital mortality in elderly COVID-19 patients were age, sex, DNR status, diastolic blood pressure, body temperature, GCS score, total bilirubin, and CRP. Most of these factors were not independently associated with in-hospital mortality in the non-elderly group except for DNR status and GCS score. Elevated CRP level was the only independent laboratory factor associated with all three outcomes, namely in-hospital mortality, endotracheal intubation, and ICU admission, in elderly patients with SARS-CoV2 infection presenting to the ED. An awareness and surveillance of these parameters may aid in triage and ED disposition decision-making and may prevent morbidities and mortality in this very important patient population.

## Supplementary Information

**Additional file 1: Table S1.** Patient characteristics by study center.

## Data Availability

The datasets generated and analyzed for this study are not publicly available but are available from the corresponding author upon reasonable request.

## Abbreviations

COVID-19: Coronavirus disease 2019
ED: Emergency department
ICU: Intensive care unit
CRP: C-reactive protein
aOR: Adjusted odds ratio
CI: Confidence interval
HFNC: High-flow nasal cannula
DNR: Do-not-resuscitate
GCS: Glasgow Coma Scale
