# Supplementary material for: Clinical factors associated with adverse clinical outcomes in elderly versus non-elderly COVID-19 emergency patients: a multi-center observational study
Source: Int J Emerg Med. 2023 Feb 22;16:11. doi: 10.1186/s12245-023-00482-4 (PMC9944782; doi:10.1186/s12245-023-00482-4)
Supplement: Supplementary file 1 — Additional file 1: Table S1. Patient characteristics by study center. [file 12245_2023_482_MOESM1_ESM.pdf]

Table S1. Patient characteristics by study center.

Characteristics	Siriraj Hospital (n=613)	Banphaeo General Hospital (n=244)	Ratchaburi Hospital (n=60)	Buddhachinaraj Hospital (n=31)	Prachuap Khiri Khan Hospital (n=30)	*p*-value
Age	65.55±16.5	56.74±19.3	60.10±18.8	61.03±21.1	46.43±15.5	<0.001
Sex (male)	303 (49.4)	111 (45.5)	43 (71.7)	15 (48.4)	20 (66.7)	0.002
Body Mass Index	25.94±6.4	26.20±6.3	24.26±4.9	23.72±4.2	27.83±6.2	0.043
Day of symptoms	4, 4	3, 4	2, 2	2, 4	2, 4	<0.001
Underlying conditions						
Coronary artery disease	77 (12.6)	10 (4.1)	1 (1.7)	1 (3.2)	1 (3.3)	<0.001
Cerebrovascular disease	82 (13.4)	4 (1.6)	4 (6.7)	1 (3.2)	1 (3.3)	<0.001
Chronic pulmonary disease	51 (8.3)	26 (10.7)	1 (1.7)	2 (6.5)	1 (3.3)	0.173
Diabetes mellitus	234 (38.2)	55 (22.5)	21 (35.0)	2 (6.5)	11 (36.7)	<0.001
Moderate to severe renal disease	128 (20.9)	8 (3.3)	8 (13.3)	0 (0.0)	1 (3.3)	<0.001
Cancer	52 (8.5)	9 (3.7)	0 (0.0)	2 (6.5)	1 (3.3)	0.019
Immuno-deficiency status	23 (3.8)	2 (0.8)	0 (0.0)	2 (6.5)	3 (10.0)	0.011
Do-not-resuscitate status	158 (25.8)	56 (23.0)	1 (1.7)	9 (29.0)	4 (13.3)	<0.001
Charlson Comorbidity Index	1, 3	0, 1	0, 2	1, 1	0, 1	<0.001
Vital signs and mental status						
Systolic blood pressure (mmHg)	140.70±30.2	127.13±25.2	136.92±23.8	135.94±24.9	127.63±25.6	<0.001
Diastolic blood pressure (mmHg)	79.51±17.5	75.74±13.5	80.15±13.7	79.90±17.5	80.80±22.2	0.035
Pulse rate (beats/min)	95.05±20.4	95.60±17.9	98.37±20.6	103.32±19.7	96.93±17.0	0.163
Respiratory rate (breaths/min)	32, 8	24, 8	24, 6	22, 7	24, 12	<0.001
Body temperature (ºC)	37.0, 1.0	36.8, 1.1	36.7, 1.4	36.9, 1.5	37.6, 1.4	0.005
Glasgow Coma Scale score	15, 0	15, 0	15, 0	15, 0	15, 0	0.113
Oxygen saturation (%)	92, 11	95, 5	92, 13	96, 6	91, 7	<0.001
Laboratory results						
Hemoglobin (g/dL)	12.5, 2.8	12.4, 2.6	13.1, 3.0	13.4, 4.8	12.2, 2.9	0.045
White blood cells count (x10^3/μL)	7.2, 4.8	6.3, 3.9	7.2, 3.4	8.9, 5.1	8.9, 4.2	0.001
Platelet (x10^4/μL)	21.6, 10.8	21.8, 9.9	19.9, 10.5	2.52, 14.5	26.4, 12.1	0.064
Creatinine (mg/dL)	1.63±2.2	1.24±1.4	1.50±1.6	1.44±1.3	1.49±2.1	
Glomerular filtration rate (ml/min/1.73m^2)	64.64±33.7	76.78±32.6	68.69±31.1	71.29±34.5	84.79±37.6	<0.001
Lactate (mg/dL)	0.52±1.5	2.81±1.2	3.5±2.9	2.38±2.9	0.97±2.5	<0.001
D-dimer (g/L)	0.67, 1.69	0.30, 0	0.45, 0.58	0, 0	0.93, 2.74	<0.001
Aspartate transaminase (mg/dL)	44, 39	47, 43	49, 34	31, 14	51, 68	0.001
Alanine minotransferase (mg/dL)	26, 28	34, 47	33, 30	26, 26	29, 40	0.014
Total bilirubin (mg/dL)	0.44, 0.36	0.51, 0.29	0.58, 0.64	0.42, 0.25	0.80, 1.00	<0.001
Procalcitonin (mg/L)	0.20, 0.55	0.41 (0.03-n/a)	0.20, 0.59	0, 0	0.17, 0.60	<0.001
C-Reactive protein (μg/L)	69.1, 91.4	70.3, 95.2	60.3, 128.4	0, 0	66.9, 122.5	<0.001
Management						
Corticosteroids	567 (92.5)	180 (74.4)	53 (88.3)	24 (77.4)	17 (56.7)	<0.001
Favipiravir	585 (95.4)	234 (96.3)	57 (95)	28 (90.3)	26 (86.7)	0.131
Remdesivir	123 (20.1)	0 (0)	13 (21.7)	7 (22.6)	2 (6.7)	<0.001
Tocilizumab	52 (8.5)	0 (0)	1 (1.7)	0 (0)	1 (3.3)	<0.001
Baricitinib	11 (1.8)	0 (0)	1 (1.7)	0 (0)	0 (0)	0.240
High flow nasal cannula	153 (25)	10 (4.1)	0 (0)	0 (0)	10 (33.3)	<0.001
Endotracheal intubation	112 (18.3)	2 (0.8)	29 (48.3)	6 (19.4)	6 (20.0)	<0.001
Extracorporeal membrane oxygenation	2 (0.3)	0 (0)	0 (0)	0 (0)	0 (0)	0.880
Inotropic drugs	108 (17.6)	2 (0.8)	25 (41.7)	5 (16.1)	0 (0)	<0.001
Renal replacement therapy	36 (5.9)	6 (2.5)	2 (3.3)	1 (3.2)	0 (0)	0.161
Emergency Department disposition						
ICU	50 (9.1)	24 (10.1)	8 (13.3)	7 (22.6)	0 (0)	<0.001
Intermediate ward	406 (66.2)	56 (23.5)	21 (35.0)	19 (61.3)	4 (13.3)	<0.001
Low-acuity ward	79 (12.9)	118 (49.6)	27 (45.0)	4 (12.9)	26 (86.7)	<0.001
[a]Hospitel	1 (0.2)	0 (0)	0 (0)	1 (3.2)	0 (0)	<0.001
Home isolation	26 (4.2)	6 (2.5)	0 (0)	0 (0)	0 (0)	<0.001
Field hospital	20 (3.3)	21 (8.8)	3 (5.0)	0 (0)	0 (0)	<0.001
Transfer	1 (0.2)	7 (2.9)	0 (0)	0 (0)	0 (0)	<0.001
Dead	24 (3.9)	4 (1.7)	1 (1.7)	0 (0)	0 (0)	<0.001
Other	0 (0)	2 (0.8)	0 (0)	0 (0)	0 (0)	<0.001
Complications						
Ventilator associated pneumonia	36 (5.9)	0 (0)	1 (1.7)	2 (6.5)	0 (0)	0.001
Hospital associated pneumonia	89 (14.5)	8 (3.3)	19 (31.7)	5 (16.1)	4 (13.3)	<0.001
Bacterial pneumonia	130 (21.2)	38 (15.6)	0 (0)	0 (0)	2 (6.7)	<0.001
Other hospital-acquired infection	88 (14.4)	1 (0.4)	0 (0)	0 (0)	0 (0)	<0.001
Septic shock	110 (17.9)	7 (2.9)	13 (21.7)	7 (22.6)	0 (0)	<0.001
Acute respiratory distress syndrome	104 (17)	34 (13.9)	5 (5.0)	5 (16.1)	0 (0)	0.016
Pulmonary embolism	26 (4.2)	2 (0.8)	1 (1.7)	0 (0)	0 (0)	0.048
Stroke	5 (0.8)	1 (0.4)	0 (0)	0 (0)	0 (0)	0.857
Myocardial infarction	5 (0.8)	0 (0)	0 (0)	2 (6.5)	0 (0)	0.002
Pneumothorax	16 (2.6)	0 (0)	4 (6.7)	0 (0)	0 (0)	0.008
Outcomes						
Hospital mortality	161 (26.3)	45 (18.4)	30 (50)	10 (32.3)	8 (26.7)	<0.001
Hospital length of stay (days)	10, 9	9, 8	13, 9	11, 9	11, 9	<0.001
ICU length of stay (days)	8, 10	5, 6	10, 6	5 (1-n/a)	12, 9	<0.001

Note: data presented as n (%), mean±standard deviation or median, interquartile range. [a]hotels that were modified into hospital for low-acuity patients.
Abbreviation: ICU, intensive care unit; mmHg, millimeters of mercury; mm^2, square millimeters.
